# Trunk Appearance Perception Scale (TAPS) discrepancy between scoliosis children and their parents influence the SRS-22 secore

**DOI:** 10.1186/1748-7161-7-S1-O3

**Published:** 2012-01-27

**Authors:** M Rigo, E D’agata, M Jelacic

**Affiliations:** 1 Institut Elena Salvá, Barcelona, Spain

## Background

The Trunk Appearance Perception Scale (TAPS) is a valid instrument to assess self perception of trunk deformity [1]. The SRS-22 has been widely used to measure Health Related Quality of Life in scoliosis population but it is not clear which factors can influence its final score [2-4].

## Study

Trunk deformity can be perceived differently by children and parents [5,6]. The aim of this study was to check whether different perception of the trunk deformity between patients and parents is a factor influencing the SRS-22 or not.

## Materials and methods

Prospective study including 71 (62 F, 9 M) patients with idiopathic scoliosis (treated and non treated), attending the clinic with their parents. Mean age 17 y ± 5.7. Mean Cobb angle 37° ± 15°. All patients completed the SRS-22 and the TAPS. Parents completed the TAPS assessing trunk deformity of their children. A coeficient of discrepancy (TAPS-CD) was defined. Statistical analysis was performed by using SPSS to compare TAPS, TAPS-CD and SRS-22.

## Results

Results showed a negative correlation between TPAS-CD and the total SRS-22 (p<.05) and treatment satisfaction (p<.05). A significant positive correlation was found between patients TAPS, self-image and pain and between parents TAPS, function and treatment satisfaction in the SRS-22. Two groups were created according to the SRS-22 score. Patients with higher score in the SRS-22 showed a higher TAPS-CD (P<.05).

## Conclusion

Discrepancy in Trunk Deformity Perception between children and parents influence the SRS-22 of the children. Patients’ perception and parents’ perception of trunk deformity influence the SRS-22 differentely.
